# Hospitalization rates and cost in severe or complicated obesity: an Italian cohort study

**DOI:** 10.1186/1471-2458-13-544

**Published:** 2013-06-05

**Authors:** Enrica Migliore, Eva Pagano, Dario Mirabelli, Ileana Baldi, Dario Gregori, Carlo Zocchetti, Cristina Tuzzi, Franco Balzola, Maria Letizia Petroni, Franco Merletti

**Affiliations:** 1 Unit of Cancer Epidemiology, San Giovanni Battista Hospital, CPO-Piemonte, CERMS and University of Turin, Turin, Italy; 2 Labs of Epidemiological Methods and Biostatistics, Department of Environmental Medicine and Public Health, University of Padua, Padua, Italy; 3 Regional Health Authority, Lombardy Region, Milan, Italy; 4 Clinical Nutrition Laboratory, IRCCS Institute Auxologico Italiano, Piancavallo, (Verbania), Italy

**Keywords:** Hospitalization rates, Hospital costs, Obesity with complications, BMI, Waist circumference

## Abstract

**Background:**

The economic and social costs of obesity are estimated to be considerable, particularly for inpatient care. The aim of this study was to compare the hospitalization rates of individuals with severe (body mass index [BMI] ≥40 kg/m^2^) or complicated (BMI ≥30 kg/m^2^) obesity with those of the general population in two regions of Northwest Italy, and to describe absolute costs of hospitalization and their determinants.

**Methods:**

Between 1996 and 2002, 6,516 patients who were admitted for the first time to a hospital offering a nutritional rehabilitation programme for obesity were enrolled and followed-up (mean follow-up time: 7.3 years). Standardized hospitalization rates (SHRs) were computed by sex for all-cause and cause-specific hospitalization. The general population of the two regions was used as the reference population. The annual cost of hospitalization was estimated for the study cohort only at the individual level, and its association with different determinants was assessed using a multivariable linear model for longitudinal data.

**Results:**

SHRs of the study cohort versus the general population increased for all-cause hospitalization (males: 3.53, 95% CI 3.45-3.61; females: 3.22, 95% CI 3.18-3.26) as well as for most obesity-related conditions. The absolute median annual cost of hospitalization was 2,436 euros for males and 2,293 euros for females. Older age at cohort enrolment, BMI ≥40 kg/m^2^, waist circumference above the median (males: 1.26 metres; females: 1.13 metres), and the presence of co-morbidities, such as cardiovascular diseases, respiratory diseases, cancer, diseases of the musculoskeletal system and connective tissue, and mental disorders, significantly increased the absolute median annual costs of hospitalization.

**Conclusions:**

The economic consequences of high hospitalization rates in obese individuals are relevant. Reducing the occurrence of co-morbidities among obese persons may be one important goal, not only for clinical reasons, but also from a public health point of view.

## Background

Obesity (body mass index [BMI] ≥30 kg/m^2^), and high-grade, or severe obesity (BMI ≥40 kg/m^2^) have been associated with increased all-cause mortality in Northern Europe [1,2] and the United States [3-6]. An Italian cohort study of individuals with severe obesity [7] showed a three-fold increase in overall mortality in males and a more than two-fold increase in females.

Obese adults are at increased risk of several diseases, including cardiovascular diseases (CVDs), diseases of the musculoskeletal system, diabetes, depression, and some types of cancer including cancers of the colon-rectum, pancreas, breast (in postmenopausal women), oesophageal adenocarcinoma, endometrium, kidney and liver, and non-Hodgkin lymphoma [8-10]. In addition to increased mortality and impaired quality of life, obesity and its associated co-morbidities may have a considerable impact on healthcare consumption. Indeed, the hospitalization rates of overweight and obese adults have been reported to be higher than those of the general population [11-13].

The economic and social cost implications of obesity are estimated to be considerable, particularly for inpatient care [14]. Obese persons consume more medication and healthcare resources, and therefore have higher costs for drugs and hospitalizations [15-18], and higher overall healthcare costs compared with normal-weight individuals [19].

The most widely used indices of body shape are BMI and waist circumference (WC) [20]. A strong association between BMI and higher healthcare consumption and costs has been reported [21-23]. Moreover, healthcare costs for a given BMI were found to be related to abdominal adiposity, as assessed by WC [24]. Due to the ever-increasing burden that obesity is putting on healthcare resources, especially for inpatient care, detailed information on the hospitalization of obese individuals may be useful in planning healthcare programmes tailored to their specific needs.

The existence of the National Health Service (NHS) in Italy offers the opportunity to study hospitalization patterns, as all Italian residents are entitled to access any NHS hospital free of charge, and without administrative limitations. Healthcare providers, both public and private, need to deliver a hospital discharge record (HDR) database in order to receive reimbursement from the NHS. All inpatient and day-care activity is then included in a regional HDR database for administrative purposes. Using the regional HDR database, the aim of this study was to compare the hospitalization rates of individuals with severe (BMI ≥40 kg/m^2^) or complicated (BMI ≥30 kg/m^2^) obesity against those of the general population, and to describe absolute hospitalization cost and their determinants.

## Methods

The study cohort included patients admitted to either the Medical Ward, or the Obesity Rehabilitation Unit of the Italian Auxologic Institute (IAI) in Piancavallo, a specialized centre for the treatment of obesity and its complications. To be admitted to the IAI, patients had to have a BMI of either ≥40 kg/m^2^, or 30–39 kg/m^2^ with complications that were not satisfactorily controlled with outpatient treatment.

The study was a clinical audit that monitored patterns of care delivered to obese patients after their discharge from the IAI. Therefore, according to the local ethical committee, at the time the study was started, ethical approval was not required. Data anonymization has been guaranteed by working with encrypted personal identification codes.

Inclusion criteria were first admission to the IAI between 1996 and 2002, age ≥18 years and residence in the Piedmont or Lombardy Region. For each cohort member, all admissions to any NHS-funded hospital after enrolment were identified from the regional HDR database by a deterministic record-linkage procedure based on tax identification number. Deaths in the study cohort were identified through the regional HDR database, in which deaths during a hospital stay are recorded. All other cohort members were assumed to be alive at the end of follow-up, i.e. 31 December 2006. Cohort members contributed person-years of follow-up from their date of enrolment (date of first admission to IAI) until date of death, or the end of follow-up.

For each individual hospitalization, the underlying cause was derived from the principal diagnosis contained in the HDR database, coded according to the International Classification of Diseases, 9^th^ revision (ICD-9). The following diagnostic categories were used: all CVDs (390–459), hypertensive disease (401–405) and ischemic heart disease (410–414), heart failure (428), all respiratory diseases (460–519), pneumonia and influenza (480–487), chronic bronchitis (491), asthma (493), all malignant neoplasms (140–208), colon-rectum neoplasms (153–154), breast neoplasms (174–175), all diseases of the musculoskeletal system and connective tissue (710–739), all mental disorders (290–319), diabetes (250), obesity (278) and rehabilitation procedures (V57). A residual category with all other diagnoses was also defined.

Diabetes was assessed by integrating all available information: ICD-9 code 250 in any diagnostic field of all available discharge records, or insulin or anti-diabetic therapy at cohort enrolment, or fasting glucose level at cohort enrolment of ≥125 mg/dl. Hypertension was considered present if ICD-9 codes 401–405 were present in any of the diagnostic fields of any discharge record.

The following clinical data and general risk factors were abstracted from the clinical records of the first admission to the IAI: height, weight, BMI and WC, systolic and diastolic blood pressure, fasting glucose, total cholesterol, low-density lipoprotein cholesterol and high-density lipoprotein cholesterol, triglycerides, tobacco smoking (any current use) and alcohol consumption. (any current consumption). Educational level and marital status were obtained from the regional HDR database.

The general population of the Piedmont [http://www.ruparpiemonte.it/infostat/] and Lombardy [http://www.asr-lombardia.it] Regions during the study period served as the reference population for the analysis of hospitalization rates (about 6.5 million inhabitants). The proportion of obese individuals in the adult population of all the regions of Northwest Italy has been estimated at around 8%-10% [25,26].

Diagnosis Related Group (DRG) tariffs, which represent the reimbursement levels of the NHS to healthcare providers, were used to estimate hospitalization costs. Tariffs from before 2001 were converted into Euros, and costs for each year were actualized to 2007 values using the general Consumer Price Index.

### Statistical analysis

Standardized hospitalization rates (SHRs) and their corresponding 95% confidence intervals (CIs) were calculated as the ratio of observed to expected hospitalizations. Observed hospitalizations did not include the admission at cohort enrolment. The expected hospitalizations were obtained by applying the age-, sex- and year-specific hospitalization rates of the reference population. SHRs by sex were computed for all-cause, and for cause-specific hospitalization, as per the diagnostic categories previously described. SHRs for diabetes (250), obesity (278) and rehabilitation procedures (V57) are not reported, as these conditions are either specific to, or very closely associated with obesity. All SHR analyses were stratified by BMI (30–39 kg/m^2^ and ≥40 kg/m^2^) and WC (below versus above the median: 1.26 m in males, 1.13 m in females), as indicators of the severity of obesity. SHRs for all-cause hospitalizations were also stratified by age group (18–29, 30–39, 40–49, 50–59, 60–69, and ≥70 years).

The absolute number of hospitalizations due to obesity was calculated as the difference between the observed and expected events. Attributable Risk (AR%) i.e., the proportion of observed hospitalizations that would have been avoided if cohort members had the same body weight distribution as the general population, was estimated as:

$$\left(\left(\mathrm{SHR}-1\right)/\mathrm{SHR}\right)\times 100$$

Individual cumulative costs of hospitalizations during follow-up were calculated by summing the DRG tariffs corresponding to the hospital stays of each cohort member. The average annual costs of hospitalization were calculated by dividing this sum by the length of follow-up in years. The association between average annual costs of hospitalization and their determinants was estimated via a multivariable Gamma Generalized Estimating Equations model with log link, assuming a first-order autoregressive correlation between costs for the same cohort member from the first to the last hospitalization. This structure is appropriate for longitudinal studies when the correlation between subsequent measurements is expected to decline with time. Estimates were weighted by the time of exposure as an offset of the logarithm of time in years and inserted in the linear predictor of the model.

Covariates included in the multivariable model, along with BMI, WC and age at cohort enrolment, were in principle all socio-demographic and clinical variables, but blood pressure, total cholesterol and triglycerides had to be excluded due to collinearity. The model also included co-morbidities identified in discharge records during follow-up. As diabetes does not usually entail inpatient treatment, and is associated with CVDs, a term was also added to model the interaction between diabetes and CVDs. All analyses were stratified by sex. Results are presented as the exponentiation of the coefficients, which can be interpreted as relative risks.

Record linkage and data management were carried out using SAS Release 8.02 (SAS Institute, Cary, North Carolina, USA). All analyses were conducted using STATA 9 (Stata Corporation, College Station, Texas, USA).

## Results

Table 1 shows the general characteristics of the study cohort. Out of 6,516 cohort members, 77.9% were females. Mean age at cohort enrolment was 48.9 years (standard deviation 14.0) for males and 51.2 years (standard deviation 14.8) for females. Half of the cohort members (53.2% females and 48.4% males) had a BMI of ≥40 kg/m^2^ (Table 1).

**Table 1:** **Socio**-**demographic and clinical characteristics of the study cohort**

	**Males**		**Females**	
	**N (1,440)**	**%**	**N (5,076)**	**%**
Age at cohort enrolment (years)				
18-29	154	10.7	558	11.0
30-39	224	15.6	622	12.3
40-49	305	21.2	882	17.4
50-59	396	27.5	1,356	26.7
60-69	281	19.5	1,171	23.1
≥70	80	5.6	487	9.6
Calendar period of enrolment				
1996-1999	210	14.6	757	14.9
2000-2003	698	48.5	2,378	46.8
2004-2006	532	36.9	1,941	38.2
Educational level				
High	559	38.8	1,421	28.0
Intermediate	596	41.4	1,783	35.1
Low	268	18.6	1,725	34.0
Unknown	17	1.2	147	2.9
Marital status				
Married	961	66.8	3,361	66.2
Other	477	33.2	1,714	33.8
	**N**	**mean (SD)**	**N**	**mean (SD)**
Height (cm)	1,440	172.2 (7.4)	5,076	157.5 (7.0)
Weight (kg)	1,440	121.3 (21.7)	5,076	102.5 (17.0)
BMI (kg/m^2^)	1,440	40.9 (6.4)	5,076	41.4 (6.6)
WC (cm)	1,402	127.4 (0.37)	5,005	113.7 (0.18)
Systolic blood pressure (mm/Hg)	1,438	142.1 (19.2)	5,038	138.9 (20.1)
Diastolic blood pressure (mm/Hg)	1,438	85.3 (10.9)	5,044	82.7 (10.5)
Fasting glucose (mg/dl)	1,432	110.1 (43.8)	5,065	100.6 (33.5)
Total cholesterol (mg/dl)	1,431	213.3 (44.2)	5,066	214.9 (43.2)
LDL cholesterol (mg/dl)	1,312	135.8 (36.2)	4,924	137.4 (50.7)
HDL cholesterol (mg/dl)	1,429	41.4 (12.3)	5,059	50.9 (13.2)
Triglycerides (mg/dl)	1,430	167 (122–235)*	5,062	128 (96–173)*
Current smoker (%)	1,416	32.1	5,002	21.2
Any alcohol consumption (%)	1,424	40.0	5,029	16.7

*median (interquartile range).

*N* number, *SD* standard deviation, *BMI* body mass index, *WC* waist circumference, *LDL* low-density lipoprotein, *HDL* high-density lipoprotein.

The mean follow-up time was 7.3 years, with 45,398 person-years of observation (10,005 for males and 35,393 for females). During follow-up 37,983 hospital admissions were identified (77.2% among females), with a median number of four hospitalizations per cohort member, regardless of sex (interquartile range: 2–7). Thirty-seven percent of all hospitalizations had a principle diagnosis of endocrine, nutritional and metabolic diseases recorded in the regional HDR database. The other most frequent diagnoses were: CVDs (9.2%), rehabilitation procedures (6.6%) and mental disorders (5.1%). A complete list of diagnoses, corresponding to the principal diagnosis recorded in the HDR database for each hospitalization, is reported in Additional file 1: Table S1.

Among males, SHRs were higher in the 30-49-year age group. In women, the highest SHR was observed in the 50-59-year age group, and it was less pronounced than among men. In both sexes, the absolute number of hospitalizations due to obesity was largest between 50 and 69 years, and BMI ≥40 kg/m^2^ and WC above the median were associated with higher SHRs (Table 2).

**Table 2:** **SHR and 95**% **CI of all**-**cause hospitalizations by sex**, **age group**, **BMI and WC at recruitment**

	**Males (py=10,004)**					**Females (py=35,393)**				
				**Hospitalizations due to obesity**					**Hospitalizations due to obesity**	
	**O**	**SHR**	**95% CI**	**O-E**	**AR%**	**O**	**SHR**	**95% CI**	**O-E**	**AR%**
**All**-**cause hospitalizations**	7,231	3.53	3.45-3.61	5,183.3	71.7	24,236	3.22	3.18-3.26	16,704.3	68.9
***Age group *( *years *)**										
18-29	362	3.69	3.33-4.10	264.0	72.9	1,357	3.31	3.13-3.49	946.4	69.8
30-39	760	5.04	4.70-5.41	609.2	80.2	2,418	2.97	2.85-3.09	1,603.3	66.3
40-49	1,065	5.03	4.74-5.34	853.2	80.1	3,180	3.46	3.34-3.58	2,259.9	71.1
50-59	2,087	4.17	4.00-4.35	1,586.5	76.0	5,929	3.81	3.72-3.91	4,373.7	73.8
60-69	2,017	3.14	3.01-3.29	1,375.5	68.2	7,012	3.43	3.35-3.51	4,965.3	70.8
≥70	940	2.11	1.98-2.25	494.8	52.6	4,340	2.43	2.36-2.51	2,555.5	58.8
***BMI *(*kg***/***m***^*2*^**)**										
30.0-39.9 plus complications	3,581	3.14	3.04-3.25	2,440.9	68.2	10,684	3.02	2.97-3.08	7,148.0	66.9
≥40.0	3,650	4.02	3.89-4.15	2,742.3	75.1	13,552	3.39	3.34-3.45	9,556.3	70.5
***WC *( *cm *)**										
≤50th percentile	3,299	3.14	3.04-3.25	2,249.8	68.2	11,468	2.97	2.92-3.02	7,605.6	66.3
>50^th^ percentile	3,698	3.93	3.81-4.06	2,757.9	74.6	12,351	3.49	3.43-3.55	8,810.2	71.3

*SHR* standardized hospitalization ratio, *95% CI* confidence interval, *py* person years; *O* observed; *E* expected, *AR%* attributable risk %, *BMI* body mass index, *WC* waist circumference (median value for males=126 cm; median values for females=113 cm).

CVDs were the most frequent cause of hospitalization in this study cohort. The proportion of hospitalizations due to obesity was particularly high for heart failure (around 81% in both genders) and for hypertensive disease (89% in males and 79% in females). Hospitalization for respiratory diseases was four-fold higher among cohort members compared to the general population. In particular, cohort members with BMI ≥40 kg/m^2^ or with WC above the median had a more than a five-fold increase in hospitalization rates. The highest SHR was found for chronic bronchitis, with a proportion of hospitalizations due to obesity of approximately 86% in both sexes (Table 3). Only females had a significant excess of hospitalizations for all malignant neoplasms. Overall, hospitalizations due to diseases of the musculoskeletal system and connective tissue were significantly more frequent in cohort members than in the general population. Obesity was also associated with mental disorders in both sexes, particularly in females with a WC above the median (Table 3).

**Table 3:** **Hospitalizations for CVDs**, **respiratory diseases**, **all malignant neoplasms**, **musculoskeletal system and connective tissue**, **and mental disorders**

	**Males (py=10,004**)					**Females (py=35,393)**				
				**Hospitalizations due to obesity**					**Hospitalizations due to obesity**	
**Causes of hospitalization (ICD-9 code)**	**O**	**SHR**	**95% CI**	**O-E**	**AR%**	**O**	**SHR**	**95% CI**	**O-E**	**AR%**
All CVDs (390–459)	1,250	3.46	3.27-3.66	888.50	71.1	2,220	2.65	2.54-2.76	1,380.70	62.3
- Hypertensive disease* (401–405)	99	5.38	4.42-6.55	80.60	81.4	339	5.35	4.81-5.95	275.60	81.3
- Ischemic heart disease (410–414)	321	2.79	2.50-3.11	206.00	64.2	328	2.42	2.17-2.70	192.50	58.7
- Heart failure (428)	228	9.11	8.00-10.4	203.00	89.0	286	4.82	4.29-5.41	226.70	79.3
Respiratory diseases (460–519)	500	4.02	3.68-4.38	375.50	75.1	1,136	3.89	3.67-4.12	844.10	74.3
- Pneumonia and influenza (480–487)	42	1.86	1.38-2.52	19.50	46.2	107	1.95	1.62-2.36	52.20	48.7
- Chronic bronchitis (491)	143	7.32	6.21-8.62	123.40	86.3	273	7.48	6.64-8.42	236.50	86.6
- Asthma (493)	18	3.74	2.36-5.94	13.19	73.3	99	4.32	3.55-5.26	76.10	76.9
All malignant neoplasms (104–208)	209	1.13	0.98-1.29	23.40	11.5	645	1.22	1.13-1.32	118.11	18.0
- Colon-rectum (153–154)	19	1.34	0.85-2.10	4.80	25.4	58	1.53	1.18-1.97	20.00	34.6
- Breast (174–175)	-	-	-	-	-	145	1.13	0.96-1.34	17.20	11.5
Diseases of the musculoskeletal system and connective tissue (710–739)	421	2.63	2.39-2.89	260.70	62.0	2,748	3.77	3.63-3.91	2,019.00	73.5
Mental disorders (290–319)	264	4.99	4.42-5.63	2,110.03	80.0	1,358	6.84	6.49-7.21	1,159.40	85.4

* Diagnosis of hypertensive diseases – ICD9 401–405 – from any of all six diagnosis codes in the HDR database.

*CVDs* cardiovascular diseases, *py* person years, *ICD-9* International Classification of Diseases, 9^th^ revision, *O* observed, *E* expected, *SHR* standardized hospitalization ratio, *CI* confidence interval, *AR%* attributable risk %.

The median and mean annual costs of hospitalization are fully reported in Additional file 1: Table S2. Higher age at recruitment, BMI ≥40 kg/m^2^, WC above the median, and the presence of several co-morbidities were all associated with an increase in average annual costs of hospitalization (Additional file 1: Table S2).

Results of the multivariable analysis are shown in Table 4. Age was associated with a small increase in costs each year in both sexes. BMI did not significantly affect average annual costs of hospitalization, whereas every 10 cm increase in WC was positively associated with an 8% increase in costs among females (Table 4). Among males the presence of respiratory diseases increased the average annual costs of hospitalization by 40% (95% CI: 10%-79%), malignant neoplasms by 25% (95% CI: 4%-51%), CVDs by 22% (95% CI: 7%-39%), hypertensive disease by 18% (95% CI: 8%-28%) and diseases of the musculoskeletal system and connective tissue by 15% (95% CI: 4%-26%). The same co-morbidities, with the exception of CVD, were significant predictors of cost also among females, even though the strengths of the associations were different between the two sexes. Among females in particular, malignant neoplasms and diseases of the musculoskeletal system and connective tissue were stronger predictors of cost, while respiratory diseases were milder predictors. Females hospitalization costs were also affected by mental disorders (16%; 95% CI: 9%-24%). The presence of diabetes was not a predictor of hospitalization costs, but cohort members with diabetes had significant increases in hospitalization costs due to CVDs in both sexes.

**Table 4:** **Determinants of average annual costs of hospitalization** (**multivariable Gamma Generalized Equations Estimate model with log link**)

	**Males**		**Females**	
**Variables**	Effect*	**(95% CI)**	Effect*	**(95% CI)**
Age (one year)	1.01	(1.00-1.01)	1.01	(1.00-1.01)
WC (10 cm)	0.97	(0.83-1.12)	1.08	(1.02-1.14)
BMI (one unit)	1.01	(0.98-1.04)	0.99	(0.98-1.00)
Hypertensive disease	1.18	(1.08-1.28)	1.21	(1.17-1.26)
Malignant neoplasms	1.25	(1.04-1.51)	1.37	(1.12-1.68)
Respiratory diseases	1.40	(1.10-1.79)	1.24	(1.10-1.38)
Diabetes	0.92	(0.82-1.03)	0.98	(0.90-1.06)
CVDs	1.22	(1.07-1.39)	0.97	(0.89-1.06)
CVDs in cohort members with diabetes**	1.27	(1.14-1.43)	1.35	(1.08-1.69)
Mental disorders	1.06	(0.61-1.85)	1.16	(1.09-1.24)
Diseases of the musculoskeletal system and connective tissue	1.15	(1.04-1.26)	1.45	(1.39-1.51)

* Effect=mean costs ratio, obtained as exponential of model coefficients. Model adjusted for height, educational level, marital status, current cigarette smoking, and alcohol consumption.

** Test for interaction: males p=0.625; females p=0.007.

*CI* confidence interval, *WC* waist circumference, *BMI* body mass index, *CVDs* cardiovascular diseases.

## Discussion

This study presents hospitalization rates in a cohort of obese, adult patients recruited in a centre specialized in the treatment of obesity and its complications, compared them to those in the general population, and estimated absolute costs of hospitalization. Hospitalization rates in this cohort were much higher than in the general population, with SHRs increasing according to the severity of obesity, as measured by BMI or WC. Approximately 70% of all hospitalizations in the cohort were attributable to obesity, and the SHRs for most obesity-related co-morbidities were significantly increased.

The results presented here on the excess hospitalization of cohort members with severe obesity compared to the general population are in line with the results of Keating et al. on outpatient services and the utilization of pharmaceutical therapies [27].

Overall, a comparatively larger effect of obesity on hospitalization rates among males compared to females was found, with the highest risk in the age groups 30–39 and 40–49 years. Among females, age-specific hospitalizations ratios showed less variation. The major difference between the sexes was in the age group 30–39 years; males had the highest and females the lowest SHRs because of different expected baseline risks, probably due to maternity-related hospital admissions in the general female population. In line with Luchsinger et al. [12] and Queensberry et al. [22], the SHR increase at older ages (>70 years) was less pronounced in both sexes, even though the absolute number of hospitalizations due to obesity remained large in females. The SHR patterns by sex and age were consistent with the corresponding patterns in standardized mortality ratios described by Mirabelli et al. [7] in a previous cohort study of patients with severe obesity.

SHRs were higher in cohort members with severe obesity and a WC above the median in both sexes, but differences by category of severity were larger among males. However, in this study cohort the presence of complications was a prerequisite for admission to the IAI for individuals with a BMI of 30–39 kg/m^2^, but not with a BMI of ≥40 kg/m^2^. Consequently, differences in morbidity between the two BMI groups were likely to have been attenuated. The capability of WC to identify patients with a worse prognosis at least as efficiently as BMI has already been described [28].

Obesity is known to be an independent risk factor for CVDs [29,30]. Many studies found a positive association between obesity and CVD-related outcomes [11-13]. In this study cohort, both sexes presented a higher number of hospitalizations due to CVDs compared with the general population (SHRs approximately 3.5 in males and 2.5 in females). No trend was observed with severity of obesity, but in this comparison the underestimation of differences due to the selection criteria applied to the study cohort may have played an important role. Particularly high SHRs (9.1 in males, 4.8 in females) for heart failure were also observed, most likely due to the well-established strain induced by obesity on heart function [29].

Obesity is associated with a wide range of respiratory conditions, including chronic obstructive pulmonary disease (COPD), asthma, obstructive sleep apnoea, pulmonary embolism and aspiration pneumonia [31]. Nevertheless, little is known about the burden of hospitalization due to obesity-related respiratory complications. Han et al. [11] found no significant difference between the number of hospital admissions due to pneumonia and COPD in obese and normal-weight patients. Kornum et al. [32] found an increased risk of pneumonia-related hospitalization among males, but the association disappeared after controlling for other chronic diseases. In this study cohort, hospitalizations for respiratory diseases were four times more frequent than in the general population. There was a clear, albeit possibly underestimated, trend in SHRs according to the severity of obesity in both sexes. COPD had the highest SHRs, while pneumonia and influenza were only slightly more frequent than in the general population.

SHRs for cancer, both overall and site-specific, were not increased in males. In females a slight excess for all cancers was observed, without any evidence of trends by BMI or WC, and a small excess for cancers of the colon-rectum specifically. The association between obesity and cancer incidence and mortality is well established [10,33-35]. However, it is important to note that this study included relatively young patients (mean age 50.7) and a limited median follow-up time of 7.3 years. Therefore, at present this cohort cannot give information on cancer hospitalization rates. Similar findings were reported in the Italian mortality cohort study [7].

Obesity is considered to be one of the most important risk factors for knee osteoarthritis [36]. A Norwegian cohort study [37] also showed a significant association between obesity and hand osteoarthritis. Weitoft et al. [13] reported a 50% higher risk of inpatient care for musculoskeletal diseases among obese patients than normal-weight individuals. In the present study cohort, the SHRs for diseases of the musculoskeletal system and connective tissue were 2.6 for males and 3.8 for females, i.e., considerably increased, even if no trend for the severity of obesity was apparent.

Finally, hospitalizations due to mental disorders were in excess in this study cohort compared with the general population, particularly among females (SHR 6.8). Several studies [38,39] reported an increased risk of mood disorders in obese individuals of both sexes. The higher SHRs among females compared to males may be attributed to an increased susceptibility to some disorders, such as bipolar disorders and social phobia [40].

SHRs for obesity, diabetes and rehabilitation were not reported for several reasons. First, these diagnoses are strongly associated with severe obesity. Second, diabetes is considered by the NHS as an inappropriate cause of hospital admission. Conversely, severe obesity can justify diabetes as a principle diagnosis in the HDR database. Consequently, SHRs for diabetes in the study cohort versus the general population could be over-estimated due to a differential attitude in filling discharge records. Third, the admissions due to rehabilitation procedures are likely to be partially covered by the first admission to the IAI.

It is important to point out that relative measures of association, such as SHRs, should be read in combination with absolute numbers to fully understand the burden of disease induced by obesity. Indeed, if considered independently, these two measures may be misleading. The hospital admissions patterns described in the present report determined a per-person mean annual cost of hospitalization of more than 3,000 euros. Since all cohort members were enrolled during a first admission to the IAI in Piancavallo, they all had at least one hospital admission, and non-zero costs of hospitalization.

The main determinants of hospitalization costs were analysed to quantify their contribution to the cost accumulation. The economic burden due to hospital use was highly affected by the presence of co-morbidities. Malignant neoplasms, respiratory diseases, hypertensive diseases and diseases of the musculoskeletal system and connective tissue were predictive of higher annual costs of hospitalization in both sexes. CVDs were a relevant cost determinant only for males, with a 24% increase in annual costs of hospitalization, whereas costs increased by 16% with the presence of mental disorders among females only.

Diabetes was not a significant determinant of hospitalization costs, but diabetes per se was expected to be a driver mainly of drug costs and outpatient care. Hospitalization of cohort members with diabetes was usually due to age and diabetes-related complications, mainly CVD. In fact, among cohort members with diabetes, CVDs were found to be a significant predictor of higher hospitalization costs in both sexes.

In the study cohort, the severity of obesity, as described by BMI, did not affect hospitalization costs. This result is in contrast to Andreyeva et al. [21], who described an increase in healthcare expenditures among moderate to severely obese males and among severely to extremely obese females. Other previous studies [19,22] on BMI and healthcare expenditures are not comparable with the observations in the present study, as they used the BMI of normal-weight patients (BMI 20–25 kg/m^2^) as a reference. The cohort selection criteria likely affected the association between BMI and costs in the present study by reducing the differences associated with BMI. WC was a significant predictor of hospitalization costs among females, with an 8% increase in costs for every 10 cm increase in WC. Previous studies suggested that abdominal adiposity may be a better predictor of healthcare costs than BMI [24,41].

The main strength of this study was that it was based on a large cohort, with baseline information available on several well-recognized risk factors for subsequent morbidity and mortality, including smoking, alcohol consumption, and various clinical parameters, which allowed us to adjust for potential confounders. However, a number of limitations must be considered when interpreting the results of this study. First, the vital status of cohort members was not systematically ascertained. Cohort members who died during a hospital stay could be identified, but all others were assumed to be living throughout the observation period. As a consequence, overestimation of the person-years in the study cohort may have occurred, and led to an underestimation of SHRs, even if the low mean age of cohort members is likely to have kept any underestimation small. Second, the cohort was enrolled at a specialized centre, and therefore should not be assumed to be representative of the overall obese population in Italy. Nevertheless, as the IAI offers a residential nutritional rehabilitation programme, but not active treatment of obesity (such as bariatric surgery) or of complications due to obesity, the impact on subsequent hospitalization patterns due to complications is likely to be limited.

Reported hospitalization rates have been standardized by age, sex and calendar year. A potential confounder of hospital use could be socioeconomic status. Nevertheless, due to the lack of information in the general population dataset, it was not possible to adjust for socioeconomic status.

Finally, costs were calculated using DRG tariffs, which are used to reimburse healthcare providers. DRG tariffs do not represent actual costs, but standard costs. While obese people have been reported to experience longer hospital stays [42], and to be likely to require more resources compared with normal-weight individuals affected by the same diseases, the DRG tariffs did not vary based on the presence of obesity, and therefore real costs were probably underestimated.

## Conclusions

The economic consequences of the high hospitalization rates of obese individuals are important. The role of obesity-related co-morbidities on cost accumulation suggests that secondary prevention programmes to reduce the occurrence of these co-morbidities may not only be beneficial to obese people, but could also control hospitalization costs. As such, further study on their economic impact is warranted. Of course, the importance of health promotion programmes aimed at preventing obesity in the general population is unquestioned.

## Competing interests

The authors declare that they have no competing interests.

## Authors’ contributions

EM and EP contributed to the study concept and design and wrote the manuscript; DM and FM contributed to the study concept and design and critically reviewed the manuscript; IB and DG contributed to the design and performance of statistical analyses; CT contributed to the data collection; CZ, MLP and FB researched data and contributed to the discussion. Each author reviewed the final version of the manuscript and approved it for publication.

## Pre-publication history

The pre-publication history for this paper can be accessed here:

http://www.biomedcentral.com/1471-2458/13/544/prepub

## Supplementary Material

Additional file 1: Table S1 Diagnoses for hospitalization (ICD-9 code). **Table S2**: Mean and median annual hospitalization costs (Euros) by selected patients’ characteristics. Click here for file
