# Supplementary material for: Hospitalization rates and cost in severe or complicated obesity: an Italian cohort study
Source: BMC Public Health. 2013 Jun 5;13:544. doi: 10.1186/1471-2458-13-544 (PMC3682879; doi:10.1186/1471-2458-13-544)
Supplement: Additional file 1: Table S1 — Diagnoses for hospitalization (ICD-9 code). Table S2: Mean and median annual hospitalization costs (Euros) by selected patients’ characteristics. [file 1471-2458-13-544-S1.doc]

**Appendix**

**Appendix** **Table I. Diagnoses for hospitalization (ICD-9 code)**

|  | **Males** | | **Females** | |
| --- | --- | --- | --- | --- |
| **Causes of hospitalization (ICD-9 code)** | **N** | **%** | **N** | **%** |
| Infectious and parasitic diseases (001-139) | 50 | 0.58 | 139 | 0.47 |
| Malignant neoplasms (140-208) | 209 | 2.41 | 645 | 2.20 |
| Benign neoplasms (210-229) | 61 | 0.70 | 310 | 1.06 |
| Other neoplasms (230-239) | 29 | 0.33 | 90 | 0.31 |
| Endocrine, nutritional and metabolic diseases, and immunity disorders (240-279) | 3,079 | 35.51 | 10,972 | 37.43 |
| Diseases of the blood and blood-forming organs (280-289) | 23 | 0.27 | 126 | 0.43 |
| Mental disorders (290-319) | 307 | 3.54 | 1,629 | 5.56 |
| Diseases of the nervous system and sense organs (320-389) | 338 | 3.90 | 1,540 | 5.25 |
| Diseases of the circulatory system (390-459) | 1,253 | 14.45 | 2,229 | 7.60 |
| Diseases of the respiratory system (460-519) | 508 | 5.86 | 1,154 | 3.94 |
| Diseases of the digestive system (520-579) | 346 | 3.99 | 1,236 | 4.22 |
| Diseases of the genitourinary system (580-629) | 288 | 3.32 | 972 | 3.32 |
| Complications of pregnancy, childbirth and the puerperium (630-677) | 0 | 0.00 | 138 | 0.47 |
| Diseases of the skin and subcutaneous tissue (680-709) | 93 | 1.07 | 277 | 0.95 |
| Diseases of the musculoskeletal system and connective tissue (710-739) | 424 | 4.89 | 2,838 | 9.68 |
| Congenital anomalies (740-759) | 23 | 0.27 | 478 | 1.63 |
| Symptoms, signs, and ill-defined conditions (780-799) | 435 | 5.02 | 836 | 2.85 |
| Injury and poisoning (800-999) | 202 | 2.33 | 703 | 2.40 |
| Supplementary classification of factors influencing health status and contact with health services (V01-V89) | 987 | 11.38 | 2,936 | 10.02 |
| Other diagnoses | 23 | 0.27 | 616 | 2.10 |
| Not known * | 16 | 0.18 | 64 | 0.22 |
| Total | 8,671 | 100.00 | 29,312 | 100.00 |

* Lack of principal diagnosis code on the HDR

**Appendix Table II. Mean and median annual hospitalization costs (Euros) by selected patients’ characteristics.**

|  | **Males (N=1,440)** | | **Females (N=5,076)** | |
| --- | --- | --- | --- | --- |
|  | **Mean (SD)** | **Median (IQ - IIIQ)** | **Mean (SD)** | **Median (IQ - IIIQ)** |
| Age at recruitment | |  |  |  |
| 18-29 yrs | 2,143 (3,036) | 1,626 (1,041-2,745) | 2,017 (2,193) | 1,625 (1,073-2,377) |
| 30-39 yrs | 2,559 (2,956) | 1,845 (1,151-3,198) | 2,270 (2,657) | 1,701 (1,164-2,725) |
| 40-49 yrs | 3,000 (3,784) | 2,131 (1,122-3,680) | 2,734 (3,193) | 2,056 (1,264-3,238) |
| 50-59 yrs | 3,613 (3,006) | 2,740 (1,616-4,527) | 3,210 (3,257) | 2,320 (1,434-4,032) |
| 60-69 yrs | 4,540 (4,260) | 3,277 (1,993-5,516) | 3,935 (3,303) | 3,036 (1,895-4,930) |
| ≥70 | 4,801 (4,288) | 4,123 (2,254-6,088) | 4,182 (2,954) | 3,367 (2,088-5,563) |
| Education |  |  |  |  |
| High | 3,153 (3,336) | 2,236 (1,260-3,989) | 2,613 (2,810) | 1,915 (1,170-3,136) |
| Intermediate | 3,381 (3,607) | 2,474 (1,453-4,120) | 3,072 (3,159) | 2,228 (1,396-3,710) |
| Low | 3,886 (4,061) | 2,790 (1,686-4,995) | 3,587 (3,247) | 2,701 (1,701-4,515) |
| Not valuable | 3,468 (3,289) | 2,929 (1,289-4,441) | 3,405 (3,716) | 2,809 (1,518-4,398) |
| Marital status |  |  |  |  |
| Married | 3,645 (3,692) | 2,671 (1,557-4,550) | 3,212 (3,193) | 2,337 (1,431-4,016) |
| Other | 2,869 (3,346) | 2,062 (1,245-3,459) | 2,947 (3,007) | 2,215 (1,380-3,676) |
| Not valuable | 992.5 (768.7) | 478.6 (478.6-1,568) | 467.5 (.) | 467.5 (467.5-467.5) |
| BMI (kg/m^2^) |  |  |  |  |
| <40 | 3,138 (3,737) | 2,200 (1,228-3,948) | 2,827 (3,076) | 1,977 (1,170-3,549) |
| ≥40 | 3,657 (3,427) | 2,707 (1,583-4,543) | 3,389 (3,165) | 2,560 (1,606-4,196) |
| WC | |  |  |  |
| Below median | 3,011 (3,604) | 2,179 (1,120-3,890) | 2,716 (2,933) | 1,963 (1,185-3,380) |
| Above median | 3,726 (3,486) | 2,719 (1,641-4,651) | 3,534 (3,280) | 2,596 (1,653-4,363) |
| Current smoker | |  |  |  |
| No | 3,509 (3,721) | 2,531 (1,470-4,359) | 3,177 (3,151) | 2,330 (1,419-3,977) |
| Yes | 3,156 (3,395) | 2,202 (1,255-3,972) | 2,888 (2,998) | 2,146 (1,369-3,471) |
| Any alcohol consumption | |  |  |  |
| No | 3,304 (3,284) | 2,528 (1,454-4,211) | 3,113 (3,132) | 2,288 (1,405-3,845) |
| Yes | 3,550 (4,062) | 2,310 (1,340-4,494) | 3,117 (2,969) | 2,313 (1,437-4,031) |
| Diabetes* | |  |  |  |
| No | 3,126 (3,453) | 2,227 (1,325-3,931) | 2,956 (2,990) | 2,176 (1,357-3,622) |
| Yes | 4,072 (3,879) | 2,936 (1,727-5,289) | 3,923 (3,595) | 2,841 (1,816-4,905) |
| Hypertensive disease (401-405)** | | |  |  |
| No | 2,469 (3,500) | 1,734 (1,052-2,966) | 2,362 (2,914) | 1,759 (1,157-2,784) |
| Yes | 3,986 (3,538) | 3,036 (1,839-4,962) | 3,717 (3,174) | 2,826 (1,732-4,767) |
| Malignant neoplasms (140-208) | |  |  |  |
| No | 3,201 (3,376) | 2,286 (1,335-3,995) | 2,994 (3,041) | 2,205 (1,370-3,692) |
| Yes | 5,730 (5,168) | 4,502 (2,759-7,191) | 4,934 (3,797) | 3,919 (2,512-6,389) |
| Respiratory diseases (460-519) | |  |  |  |
| No | 2,964 (3,320) | 2,159 (1,261-3,706) | 2,847 (2,882) | 2,137 (1,346-3,484) |
| Yes | 5,119 (4,141) | 4,139 (2,411-6,147) | 5,145 (4,039) | 4,101 (2,518-6,466) |
| CVD (390-400; 406- 459) | | |  |  |
| No | 2,709 (3,300) | 1,978 (1,188-3,336) | 2,741 (2,913) | 2,041 (1,283-3,305) |
| Yes | 4,689 (3,787) | 3,677 (2,253-5,728) | 4,414 (3,494) | 3,449 (2,157-5,531) |
| Mental disorders (290-319) | |  |  |  |
| No | 3,359 (3,448) | 2,359 (1,381-4,222) | 3,046 (3,071) | 2,242 (1,389-3,777) |
| Yes | 3,649 (4,716) | 3,278 (1,656-4,284) | 3,513 (3,416) | 2,622 (1,520-4,376) |
| Diseases of the musculoskeletal system and connective tissue (710-739) | | | |  |
| No | 3,185 (3,632) | 2,221 (1,284-3,998) | 2,660 (3,104) | 1,921 (1,240-3,099) |
| Yes | 4,150 (3,372) | 3,239 (2,029-5,247) | 4,175 (2,947) | 3,379 (2,189-5,369) |
|  |  |  |  |  |
| Overall | 3,389 (3,599) | 2,436 (1,405-4,242) | 3,125 (3,136) | 2,293 (1,413-3,922) |
|  |  |  |  |  |

* Diagnosis of diabetes – ICD9 250 – from any of six diagnostic codes in the Hospital Discharge Records database, insulin or anti-diabetic therapy at enrolment, baseline fasting glucose level ≥125mg/dl.

** Diagnosis of hypertensive diseases – ICD9 401-405 – from any of all six diagnosis codes in the Hospital Discharge Records database.
